# At the heart of the matter: how mental stress and negative emotions affect atrial fibrillation

**DOI:** 10.3389/fcvm.2023.1171647

**Published:** 2023-06-20

**Authors:** Donato Giuseppe Leo, Hizir Ozdemir, Deirdre A. Lane, Gregory Y. H. Lip, Simon S. Keller, Riccardo Proietti

**Affiliations:** ^1^ Liverpool Centre for Cardiovascular Science at University of Liverpool, Liverpool John Moores University and Liverpool Heart & Chest Hospital, United Kingdom; ^2^ Department of Cardiovascular and Metabolic Medicine, Institute of Life Course and Medical Sciences, Faculty of Health and Life Sciences, University of Liverpool, Liverpool, United Kingdom; ^3^ Danish Center for Clinical Health Services Research, Department of Clinical Medicine, Aalborg University, Aalborg, Denmark; ^4^ Department of Pharmacology and Therapeutics, Institute of Systems, Molecular and Integrative Biology, Faculty of Health and Life Sciences, University of Liverpool, Liverpool, United Kingdom

**Keywords:** atrial fibrillation, cardiac autonomic nervous system, mental health, psychological stress, stress

## Abstract

Atrial fibrillation (AF) is the most common form of cardiac arrhythmia, affecting 2%–3% of the world's population. Mental and emotional stress, as well as some mental health conditions (e.g., depression) have been shown to significantly impact the heart and have been suggested to act both as independent risk factors and triggers in the onset of AF. In this paper, we review the current literature to examine the role that mental and emotional stress have in the onset of AF and summarise the current knowledge on the interaction between the brain and heart, and the cortical and subcortical pathways involved in the response to stress. Review of the evidence suggests that mental and emotional stress negatively affect the cardiac system, potentially increasing the risk for developing and/or triggering AF. Further studies are required to further understand the cortical and sub-cortical structures involved in the mental stress response and how these interact with the cardiac system, which may help in defining new strategies and interventions to prevent the development of, and improve the management of AF.

## Introduction

Atrial fibrillation (AF) is the most common arrhythmia, affecting 2%–3% of the population globally, is characterized by the presence of a rapid and irregular beating of the heart's atrial chambers (1). Common risk factors related to incident AF are hypertension, excessive/binge alcohol consumption and smoking, and the presence of other conditions such obesity, chronic obstructive pulmonary disease (COPD), coronary artery disease, congenital heart disease, sleep apnea, diabetes mellitus and thyrotoxicosis (2–5). The current management of AF has moved towards toward more holistic or integrated care approach, formulated as the ABC (Atrial Fibrillation Better Care) pathway (6), following appropriate evaluation and characterisation of the AF (7). This holistic approach is recommended in international guidelines (8).

Mental stress is an epidemic in today's society, with more than 74% of people reporting to be overwhelmed or unable to cope with daily life (9). Moreover, the recent COVID-19 pandemic has further affected the mental wellbeing of the general population (10). Psychological factors such as stress and mental disorders can affect cardiac arrhythmias and AF due to neuroendocrine (hypothalamic-pituitary-adrenal axis) and nervous (autonomic nervous system) system responses (11–14). Excessive stress can affect both frequency of arrhythmias as well as the impact of ventricular fibrillation (14). Mental health disorders may also play a role in the onset of cardiac arrhythmias (13, 15, 16).

Anxiety has been proposed as a possible trigger of cardiac arrhythmias including AF due to increased sympathetic tone and reduction of vagal tone (16). Interestingly, depression has been linked to increased risk of ventricular fibrillation and thus of sudden cardiac death (SCD), especially in patients with coronary artery disease (15). In a systematic review examining the prevalence of depression and anxiety in patients with AF, results showed that one third of AF patients have high level of depression and anxiety, negatively affect their overall quality of life (17).

The objective of this review is to summarise the current understanding on the pathophysiological brain-heart interaction, focusing on the role that psychological factors (such as negative emotions or mental disorders—e.g., depression) have on the development and progression of AF.

## Atrial fibrillation and mental stress

Mental stress has negative effects on the heart and on the cardiovascular system in general (18, 19). Episodes of acute stress increase blood pressure (20), and recurrent episodes (chronic stress) may induce damage to the endothelium which increases the risk for cardiovascular events such as stroke and myocardial infarction (21). The likelihood of incident cardiac arrhythmias is strongly associated with abnormalities in electrical repolarization of the heart, where mental stress plays a role in influencing the electrical activity (22), causing increased heart rate, decreased PR and QT interval, and a prolonged QTc interval.

The role that mental stress has in altering left atrial electrophysiology (*P*-wave axis), a known marker of AF risk, has been observed in two studies (23, 24). In both these pre-post studies in patients with stable coronary heart disease, acute stress (administered via the speech tasks test) was associated with the development of abnormal *P*-wave axis (Table 1) (23, 24). Although these studies did not assess the occurrence of AF, the ECG changes during mental stress may represent a hallmark for stress-induced alteration in the left atrial electrophysiology which may trigger AF.

**Table 1:** Summary of studies investigating the role of mental stress and emotions on atrial fibrillation.

First author, year, country	Design, study population	Outcome(s)	Mental stress test/procedure	Results	Conclusion
Feng, 2020 Norway	Prospective large population-based study, *N* = 37,402 participants (mean age 53.4 15.2 years, 56.5% women), mixed population	Depression (HADS), Incidence of AF	N/A	No significant association between anxiety or severe depression and incident AF (adjusted^c^ HR: 0.9; 95% CI: 0.6 to 1.3)	Mild to moderate depression symptoms increases the risk of developing AF Neither anxiety nor severe depression seems to increase the risk of AF
Almuwaqqat, 2020 USA	Observational study, *N* = 359 participants (mean age 56 ± 9.9 years, 62% male) with coronary artery disease	Changes in ECG	Speech Task	Risk of developing an abnormal P-wave axis (OR = 1.37; 95% CI: 1.03 to 1.30)	Acute psychological stress can affect the electrophysiology of the heart and can predispose to AF
Garg, 2019 USA	Multicentre longitudinal community-based study, *N* = 6,664 participants (mean age 62 ± 10 years, 53% female) with depressive symptoms at baseline and without previous AF	Depression (CES-D), incidence of AF	N/A	Depressive symptoms increased the risk of developing AF (HR: 1.34; 95% CI: 1.04 to 1.74), but no there was no significant association between anger, anxiety or chronic stress and onset of AF	Depression is associated with a higher risk of developing AF
Soliman, 2017 USA	Population-based Prospective cohort, *N* = 8,812 (mean age 58.1 ± 7.8 years, 63.2% women) with hypertension, dyslipidaemia and diabetes	Incidence of AF	N/A	Emotional stress due to involuntary unemployment is associated with an increased risk of AF (OR:1.54; 95% CI: 1.04, 2.37)	Involuntary unemployment is associated with a higher risk of AF
O’Neal, 2017 USA	Pre-post study, *N* = 422 patients (mean age = 56 ± 10 years, 61% men) with stable coronary heart disease	Changes in ECG	Speech Task	P-Wave terminal force in lead V_1_ (mean change = −348, 95% CI = −515 to −182)	Acute mental stress alters the electrophysiology of the left atria
Kivimäki, 2017 UK, Denmark, Sweden, and Finland	Population-based Prospective cohort *N* = 85,494 working people (mean age 43.4, range 17–70 years, 34.5% men)	Incidence of AF	N/A	Psychological stress due to prolonged working hours is a risk factor in developing AF (HR: 1.42: 95% CI: 1.13 to 1.80)	Individual that work for long-hours may have an increased risk of developing AF
Fransson, 2015 Sweden	Population-based Prospective cohort; N = 13, 477 working people (mean age 47.4 ± 10.8, 45.3% men)	Incidence of AF	N/A	Job strain was significantly associated with the risk of developing AF (HR:1.93; 95% CI: 1.10 to 3.36)	Work-related stress may be a risk factor for AF
Lampert, 2014 USA	Case-control crossover study, *N* = 95 AF patients (age and gender distribution not reported)	AF episodes, reported emotion (eDiary)	Use of eDiary to note the emotions of the day	• Patients more likely to report an episode of AF after reporting sadness (adjusted^b^ OR: 5.39; 95% CI: 3.20 to 9.75), anger (adjusted^b^ OR: 4.46; 95% CI: 2.38 to 8.36) and stress (adjusted^b^ OR: 3.07; 95% CI: 1.53 to 6.13) • Likelihood of reporting an event of AF was 85% lover after reporting of positive episodes such as happiness (adjusted^b^ OR: 0.12; 95% CI: 0.06 to 0.22)	Negative emotions trigger AF
Cheng, 2013 Taiwan	Observational, *N* = 3,888 with Panic disorder (mean age 42.21 ± 15.02 years, 36.2% men) and *N* = 3,888 without Panic disorder (mean age 46.35 ± 15.01 years, 36.0% men)	Onset of AF	N/A	Panic disorder may be classified as an independent risk factor for the development of AF (adjusted^a^ HR: 1.73; 95% CI: 1.26 to 2.37)	Panic disorder is associated with increased risk of AF
Whang, 2012	Population-based Prospective cohort *N* = 30,746 female healthcare professionals	Onset of AF; Psychological symptoms (Mental Health Inventory-5)	N/A	No significant association between psychological distress and AF (HR = 0.99; 95% CI: 0.78 to 1.25) or between depression and AF (HR = 0.99; 95% CI: 0.78 to 1.25) in this population	Global psychological distress and depression are not linked to AF onset
Tully, 2011 Australia	Observational; Patients undergoing cardiac surgery, *N* = 224 (non-AF group: mean age 61.6 ± 9.7 years, 16.5% women; post-operative AF group mean age 67.7 ± 8.3 years, 17.9% women)	New onset of AF; Depression and anxiety (DASS)	N/A	Post-operative anxiety was associated with increased risk of experiencing episodes of AF (OR: 1.09; 95% CI:1.00 to 1.18)	Anxiety showed to be associated with increased odds of post-operative AF
Mattioli, 2008 Italy	Case-control; Patients with first episode of AF, *N* = 400 (mean age 54 ± 11 years, 51% men)	Life Changes Scale, Number of cups of espresso coffee, alcohol consumption, chocolate consumption, BMI, waist-to-hip ratio	N/A	Stress-induced lifestyle changes as risk factors for AD (OR: 2.14; 95% CI: 0.29 to 3.20)	• Acute stress induces higher risk of AF; • High espresso coffee consumption and obesity are associated with increased risk of persistent AF
Lange, 2007 Germany	Prospective observational; AF patients undergoing cardioversion N = 54 (mean age 66.1 ± 9.0 years, 32% women)	Depression and anxiety (HADS), AF recurrence, LA enlargement and LV dysfunction	N/A	Depressed mood was associated with increased risk of recurring AF within 2-months of cardioversion (OR = 8.6; 95% CI: 1.7 to 44.0)	Depression as increased risk factor for AF recurrence after cardioversion
Eaker, 2005 USA	Population-based Prospective cohort (Framingham offspring); *N* = 3,682 (mean age 48.5 ± 10.1 years, 52% women)	10-year incidence of CHD, AF and total mortality	N/A	Anger and anxiety are an independent factor in developing AF in men (respectively RR: 1.28; 95% CI: 1.08 to 1.52; and RR: 1.16; 95% CI: 1.01 to 1.33)	• Tension as increased factor for CHD, AF and mortality in men; • Anxiety increased risk factor for mortality in men and women
Lévy, 1999 France	Case series, *N* = 756 patients (mean age 68.6 11.4 years, 58% men) with AF (of which 167 with PAF)	Recurrence of AF (for PAF), Complications (e.g., heart failure); death	N/A	Of the 167 patients with paroxysmal AF, 31.3% had a recurrence, which was attributed to exercise or mental stress in 10 cases	Mental stress and exercise were responsible for recurrence of AF in 6% of patients with PAF.

AF, atrial fibrillation; BMI, Body Mass Index; CI, Confidence Interval; CES-D, Center for Epidemiologic Studies Depression Scale; CHD, coronary heart disease; DASS, Depression and Anxiety Stress Scale; ECG, Electrocardiogram; HADS, Hospital Anxiety and Depression Scale; HR, Hazard Ratio; N/A, Not applicable; OR, Odds Ratio; RR, Relative Risk.

^a^ Adjusted for diagnosis of panic disorder, age, male sex, hypertension, history of coronary artery disease, diagnosis of congestive heart failure, diagnosis of valvular heart disease.

^b^ Adjusted for age, sex, use of beta-blockers, simultaneous alcohol intake, day of week (weekday/weekend), and season.

^c^ Adjusted for age, sex, weight, height, smoking, occupation type, marital status, physical activity level, alcohol consumption, presence of chronic disorders.

Two additional studies (25, 26) have investigated the role that mental stress has in triggering recurrent AF. Of particular relevance is a cross-over study (25) that investigated the role of negative emotions in triggering AF in a cohort of 95 patients with intermittent-persistent or paroxysmal AF, and provided patients with 24 h Holter-monitoring and an electronic diary to report the experience of positive and negative emotions daily for one year. The results showed that negative emotions are a trigger for AF, with most patients more likely to experience an episode of AF after reporting sadness, anger and stress; while the likelihood of an AF event was 85% lower after reporting of positive emotions, such happiness. Acute stress has also been identified as trigger for the recurrence of AF (26), in a prospective study of 756 patients with AF (of which 167 had paroxysmal AF) with a mean follow-up of 8.6(±3.6) months. Of the 167 patients with paroxysmal AF, 31.3% had a recurrence, which was attributed to exercise or mental stress in 10 cases.

Mental stress, mental exhaustion and negative emotions have been identified as risk factor for recurrent AF (Table 1) (27–31). A case-control study (28) reported that a high level of acute stress induces lifestyle changes (e.g., consumption of more coffee) and was identified as a risk factor for AF. Job strain (30, 31) (a measure of work stress) and prolonged working hours (29) have been both identified as risk factors for AF. Additionally, the stress due to involuntary unemployment has also been associated with an increased risk of AF occurrence (32). However, a prospective cohort study (32–33) observing female health professionals showed no significant association between psychological distress and AF in this population. Moreover, anger and anxiety have been reported as independent risk factors for AF (34–36), especially for men. However, a retrospective study (36) on a total of 3,888 patients with panic disorder and without a diagnosis of AF found no significant association between anger, anxiety or chronic stress and onset of AF, however, depressive symptoms increased the risk of developing AF. Depression has also been associated with recurring AF in another prospective cohort study (37) observing 54 patients with persistent AF undergoing electrical cardioversion, but not for incident AF (38, 39).

The quality of studies examining the relationship between mental stress and incident AF or recurrent AF varied markedly, in the quantification of mental stress (using different scales/tests), varying sample sizes, and most studies were observational in nature, with varying lengths of follow-up. To date, only one study (25) has investigated negative emotions as a trigger of episodes of AF in a crossover study design. Therefore, more studies are needed to further understand the relationship between emotions, mental stress and AF, especially the role these factors have in triggering AF. Stress biomarkers (e.g., catecholamines) and ECG readings in response to acute stress, as well as the standardisation of scales and questionnaires to assess chronic stress in this population should be assessed in large clinical trials investigating the role that emotions and stress (both acute and chronic) have in triggering AF.

## Role of the immune response

It is well known that the body stress response triggers inflammatory mechanism (e.g., increasing of circulating inflammatory cytokines) (40). Chronic stress has been associated with an increased likelihood of developing cardiovascular diseases (e.g., coronary heart disease, stroke) (41), due to the inflammatory response that it activates in the body (40, 42). This is also true for the development of arrhythmias, with the later also promoting inflammation itself, leading to a cycling interaction between inflammation and disease (43). Several studies (44–47) have associated AF with increased plasma interleukins (IL-6; IL-1ϐ), suggesting the role of inflammation in the developing of AF after cardiac surgery. The role of neuroinflammation (e.g., inflammation of the brain areas related to sympathetic output) in the developing of neurogenic hypertension (48) validated further the role that inflammation has in increasing risks factors for AF. Furthermore, the increased peripheral cytokines release (such as TNF-ɑ) after acute myocardial infarction (which is commonly followed by the onset of AF), induces an increased permeability of the Blood Brain Barrier and a long persisting neuroinflammatory status that emphasize the relationship between stress, inflammation and onset of AF (49).

## Heart-brain interactions

Autonomic and neurohumoral control of cardiovascular function is under the control of the central nervous system (50). Heart-brain interactions have a number of manifestations, such as the so-called “stroke-heart” syndrome, where neurological deficits induced by an ischaemic stroke affect the cardiovascular system (51). For example, a recent retrospective cohort study showed that following stroke, new-onset cardiovascular complications are very common and associate with a worsening prognosis of major adverse cardiovascular events (52).

**Figure 1:**
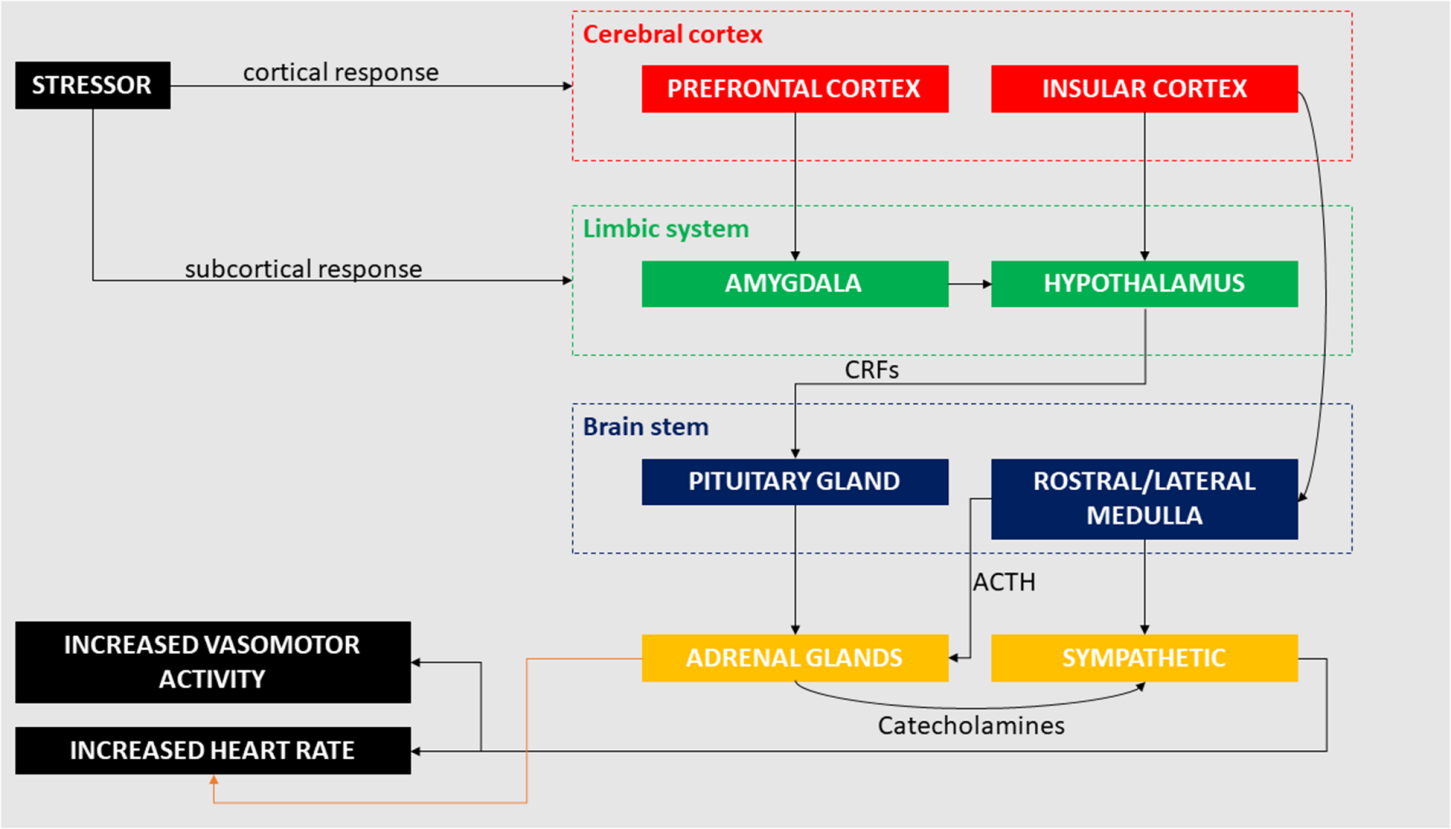
Cortical and subcortical pathways involved in the cardiac response to mental and emotional stress. Stressors (e.g., negative emotions, job strain) can activate conscious response through the pre-frontal cortex as well as unconscious response through the amygdala, both inducing changes in cardiac activity.

The interaction between brain and heart in terms of cerebral influence on cardiac output has been investigated in several studies, which have focused on underlying pathophysiological processes (50, 53–72). Cerebral damage (e.g., following stroke) has been linked to the occurrence of myocardial infarction and arrhythmias, which suggests a major role of the central nervous system in regulating cardiac functions (50, 55). In recent years, the link between the heart and brain after stroke has been further described in the already mentioned “stroke-heart syndrome” (51), which induces autonomic dysfunctions resulting in reduced heart rate variability and impaired baroceptor reflex sensitivity. This post-stroke induced autonomic dysfunction is exaggerated during sleep (59). Effects of haemorrhagic stroke (i.e., subsequent subarachnoid haemorrhage) causes ECG changes such as ST-elevation or depression and QT prolongation (56). These changes have been linked to increased risk of developing AF following haemorrhagic stroke (50, 64). Dysfunction in any cortical or subcortical brain system may lead to pathological changes in the cardiovascular system (50) and cardiac abnormalities are associated with multiple neurological disorders other than stroke and haemorrhage, including brain (62, 63) and spinal cord injury (58, 65), epilepsy (66, 67, 70, 72), neurodegenerative diseases (57, 60, 68), migraine (53, 61), and sleep disorders (54, 71). It is now evident that cortical and subcortical areas of the brain interact with peripheral structures (e.g., adrenal glands) that activate a series of physiological responses which ultimately affect the heart (69) (Figure 1).

The role of neurotrophins (e.g., Brain-Derived Neurotrophic Factor—BDNF) in the brain health and development have been largely investigated (73). Several studies have also reported the crucial role of neurotrophins in non-neuronal cells (74–76), and their effects on the neuronal regulation of heart functions (76). BDNF has shown to have a protective role on the health of the heart, promoting angiogenesis and neovascularization of ischemic tissue through the recruitment of endothelial cells and by regulating their survival (77), with high serum level of BDNF being associated with a lower risk of cardiovascular disease (78).

Areas of the brain underlying individual perception of emotional stress include a network of cortical areas and subcortical nuclei. Subcortical networks have long been acknowledged to regulate stress and influence the cardiovascular response and more recently it has become apparent that the cardiovascular system is in part regulated by modulation of the cerebral cortex (79). At the cortical level, mental and emotional stress is principally regulated by the prefrontal cortex (PFC) and insular cortex.

The PFC has wide-ranging important roles in many aspects of higher order cognitive and affective functions given its extensive neural connections with other cortical and subcortical brain regions. These functions include, but are not restricted to, memory and language functioning, emotional processing, behavioural inhibition, social behaviour, personality expression, decision making, modulation of autonomic responses, and sensory integration, amongst others (80–88).

During a stressful event, the medial and orbital PFC (and cingulate cortex) plays a role in moderating the stress response via connection with limbic structures (in particular, the hippocampus and amygdala) that are involved in the neuroendocrine response to stress (89), and which may affect acute and chronic-stress induced cardiovascular response (90). Both acute and chronic stress directly impact on brain structure and function.

Acute mild stress can cause impairment of cognitive abilities mediated by the PFC and sustained chronic emotional stress may lead to damage to prefrontal neurons (91). Unlike other brain regions, even short periods of emotional stress can cause architectural changes to prefrontal neurons (92, 93). High levels of catecholamine release during stress have a deleterious impact on PFC functioning and strengthens the emotional and habitual responses of the amygdala and basal ganglia (94). Patients with heart failure have reduced grey matter density of the medial prefrontal cortex (amongst other regions), which correlates with N-terminal prohormone of brain natriuretic peptide—a biomarker of heart failure (95). Young patients with congenital heart defects have reduced volumes of the lateral, medial, and orbital PFC and concomitant cognitive deficits (96). Patients with vascular risk factors but without clinically manifest cardiovascular or cerebrovascular disease or events show evidence of prefrontal (and other regional) brain abnormalities (96). Resting-state functional MRI work has reported that temporal changes of heart rate variability are correlated with dynamic changes of PFC connectivity and that heart rate variability biofeedback leads to a drop in heart rate and concomitant increase in functional connectivity between PFC and amygdala, insula, and cingulate cortex (97). Animal studies have revealed that emotionally stressed animals with knockdown levels of glutamatergic packaging in prefrontal interneurons have increased heart rate and mean arterial pressure reactivity relative to unstressed controls (90). Further studies on animal models have highlighted the negative role that psychosocial stress has in deteriorating both cardiac structure and function in arrhythmogenic cardiopathy mice, showing an increased risk of sudden death (98).

The insular cortex shares reciprocal neural connections with PFC, amygdala and other limbic areas, acting as a hub linking large-scale brain networks, and has important roles in various sensory, emotional, motivational, and cognitive functions (99–101). The insular cortex also plays an important role in the central control of cardiac functions as it acts directly on the autonomic nervous system (103, 104), and is consistently implicated in stress-related social and anxiety disorders (105, 106). Recent work has demonstrated a site-specific regulation of cardiovascular stress response along the rostro-caudal axis of the insular cortex (107). There is also accumulating evidence indicating that acute stress impacts on the normal organisation of resting-state functional brain networks (108, 109), which include prefrontal and insular hubs, and which may serve to enable efficient coping (108). Moreover, given that the insula is proximal to the middle cerebral arteries, it is exposed to higher risk of cerebrovascular disease: insula damage typically from stroke has been associated with a multitude of cardiac complications including arrhythmia, diurnal blood pressure variation, myocardial injury, and increased brain natriuretic peptide, catecholamine, and glucose (79, 110). A significantly higher prevalence of previously undiagnosed AF is associated with stroke that impacts the insula compared to stroke that spares the insular cortex (111) and acute stroke of the insula can lead to heart failure (112). Heart rate and blood pressure changes have been reported in response to human insular stimulation (113) and there is a correlation between insula (and extra-insular) activity recorded using functional neuroimaging and heart rate variability (114–116).

Subcortical regions regulating the stress response include components of the limbic system proper and peri-limbic areas, especially including the amygdala, hippocampus and hypothalamus, and brainstem regions, including the peri-aqueductal grey (117). Subcortical pathways that involve the limbic system may regulate the body's response to mental and emotional stress, with and without cortical interaction. The amygdala and hypothalamic-pituitary-adrenal (HPA) axis play important roles in regulating the neuroendocrine response to stress (118) and can trigger the stress-mediated response without cortical initiation (89). Under a stressful situation, the amygdala sends distress signals to the hypothalamus which, through the release of corticotropin-releasing factors (CRFs), induces the pituitary gland to release adrenocorticotropic hormone (ACTH) into the bloodstream. ACTH targets the adrenal glands, which release cortisol and catecholamines in response (119). The increased level of catecholamines in the blood stream induces a series of cardiovascular effects that include increased heart rate, peripheral vasoconstriction and increased cardiac output, affecting in general the sympathetic nervous system (120). Increased adrenergic activity has been observed in the minutes preceding AF (121). Effects induced by catecholamines on the heart affect the sinoatrial node (SAN), the pacemaker of the heart, shortening the diastole and thus increasing the heart rate (122). The influence of catecholamines in promoting atrial arrhythmic activity may have a role in the onset of cardiac arrhythmias (122). Amygdala resting activity assessed using ^18^F-fluorodexoyglucose PET, a marker of neural glucose metabolism, was reported to be significantly related to bone-marrow activity, arterial inflammation and risk of cardiovascular disease events (123). In the same study, perceived stress was associated with amygdala resting glucose metabolism (123). In a separate ^18^F-fluorodexoyglucose PET study, amygdala activity was associated with major adverse cardiac events and lower socioeconomic status, further suggesting a strong link between social stress, regional brain function, and cardiac pathology (124). MRI studies have also indicated abnormal structural and functional limbic (e.g., amygdala hyperactivity, hippocampal atrophy) findings in people exposed to chronic stress (125, 126). Hippocampal atrophy has been reported in patients with heart failure (95, 127) and stroke-free patients with AF (128). Other work has reported significantly reduced total brain volume and lower memory scores in older non-demented patients with AF (129–131), particularly in those with an increasing burden of arrhythmia (129). Moreover, brain MRI perfusion studies reveal that persistent AF decreases blood flow to the brain and perfusion of brain tissue (132).

Furthermore, the interactions between the central and peripheral nervous system in response to stress, and how it affects the cardiovascular system are highly relevant. The autonomic nervous system (ANS) is a component of the peripheral nervous system designated to regulate involuntary physiological responses (e.g., heart rate; digestion) (133). It is further divided in sympathetic (SNP—which regulates the “fight or flight” response), parasympathetic (PNS—which promotes a more generic rest status), and enteric (ENS—that regulates the digestion process) (133). Under stress, the increased sympathetic activity influences the heart response, including acceleration of the heart rate, reduction of venous capacitance, increasing of cardiac contractility and peripheral vasoconstriction (134). Negative emotions cause a release of catecholamine, which increases sympathetic tone and decreases parasympathetic tone (135). In AF, the arrhythmia can be triggered by both sympathetic and parasympathetic activation (136), where patients with structurally normal hearts are more likely to show AF following vagal activation (e.g., post-exercise AF), and patients with structural heart disease more likely to show AF after sympathetic activation (e.g., during exercise) (137). In some cardiovascular conditions sympathetic activation can trigger ventricular arrhythmia and sudden cardiac death (138). Sympathetic hyper-innervation (nerve spouting) has been linked to ventricular tachycardia and ventricular fibrillation in animal models (139).

Undoubtedly, an elevated sympathetic output is the final effector of the emotional response. Its' integrated cortical and subcortical control is complex, involving the nucleus solitarius, hypothalamus and rostral ventrolateral medulla (140, 141). Of note, two relevant features of this emotional response pathway have been described: first, the activation of the sympathetic system can be triggered by external stimuli through the amygdala and hypothalamus without involvement of the cerebral cortex (140, 141). Second, the sympathetic baroreflex response is disjointed from the sympathetic activation triggered by emotional stress.

It is also important to note the bidirectional nature of the interaction brain-heart, and how the latter can influence the former, such as inducing behavioural status (e.g., anxiety-related behaviour) following peripheral physiological changes (e.g., increasing in heart rate) (142). Heart failure has shown to increase the risk for cognitive decline (143), mainly due to the induced reduction in the brain perfusion, with decreased cardiac function also changing the level of inflammatory markers in the brain (144). Considering the heart-brain axis (145). dysfunction affecting either the heart or the brain, does reflect on the other organ, creating an interconnected loop.

## Can the reduction of mental stress be a preventive strategy for the recurrence of atrial fibrillation?

Psychological interventions, such as cognitive behavioural therapy, have been suggested to mitigate the effects of stress and mental health disorders on cardiovascular conditions (e.g., coronary artery disease) (146, 147). It is clear from accumulating evidence that psychological stress and negative emotions play an important role in the development of cardiac arrhythmias, and more specifically in the onset of AF (14, 16). Despite guidelines highlighting the role that psychological distress has on AF (148), the identification and prevention of mental and emotional stress is not always targeted in clinical practice and its clinical management remains elusive.

Acting on psychological stress factors (e.g., prolonged working hours) and on mental wellbeing (e.g., reduction of anxiety) can potentially reduce the incidence and recurrence of AF and positively impact the overall healthcare costs of this condition (149). Indeed, the major cost driver in the management of AF is due to hospitalization (150). It is then clear than preventing AF and reducing the recurrence of hospitalization may positively impact the healthcare costs. However, interventions that act on the patient's individual response to stress are currently difficult to implement in clinical practice due to the still widespread misconception that stress cannot be systemically identified and measured (151). Suggestions on how to assess both stressor exposure and stress response have been highlighted in the literature (151), and appropriate education needs to be provided to healthcare professionals on the topic. This may help with the design of future studies aiming to investigate the role that psychological interventions may play in the management of AF and may also reduce associated healthcare costs.

Stress reduction is a complex topic that needs to consider societal demands (such as number of working hours (31) or unemployment related stress (32), emotion-driven reactions (35) and the individual level of stress and mental health (17). The additional impact that stress has on the individual's lifestyle also has to be taken into consideration, as it may lead to the increase of other risks factors related to AF (28). From a clinical perspective, the patient's level of stress, as well as their mental health should be ascertained as standard during clinical consultations, with appropriate counselling and stress-reduction interventions such as cognitive behavioural therapy, mindfulness or yoga (152–154), where required. Additionally, the psychological distress of living with chronic conditions (155) should also be taken into consideration and addressed with appropriate educational intervention and directing the patients to discuss their worries with health professionals where needed.

In cases where psychological interventions are needed, their implementation in clinical practice may face several limitations which need to be addressed. Appropriate length and frequency of intervention, as well as the modality of administration (e.g., in person, online) should be properly investigated considering the specific needs of patients with AF. Additionally, the type of intervention should be evaluated and tailored for the specific psychological risk factors (e.g., stress, anxiety, depression). These considerations would need to be investigated in randomised controlled trials before defining appropriate guidelines for healthcare professionals.

Further investigation is also required to better understand the heart-brain interaction. Studies involving brain imaging and cardiac measurements (e.g., ECG) under laboratory-induced mental stress are required that differentiate the cerebral and cardiac response during both mental stress and emotional precipitators (e.g., anger, anxiety).

## Future perspectives

Traditionally the role of the autonomic nervous system in the onset of AF has been well acknowledged. Current evidence highlights cases of sympathetic driven AF, in which episodes of arrhythmia develop in response to physical activity or emotional events, and cases of parasympathetic driven AF, in which episodes are triggered by binge eating or drinking, as well chocolate assumption (136). However, the significance of such findings is currently confined to anecdotal evidence and is difficult to translate into clinical practice. As part of the holistic management of AF, attention to psychological morbidity associated with AF is also needed, as suggested by the Atrial Fibrillation Better Care pathway (6, 156). Appropriate recognition by the cardiology community of the role that psychological factors play in the onset and progression of AF should be highlighted to promote assessment of these factors during clinical consultations and to provide empirical evidence for their impact on the management of patients with AF.

Further research should address the current lack of mental health screening in the routine clinical assessment of patients that are potentially at risk of developing AF, or of patients that are currently under management for AF. Appropriate screening methods should be defined and validated, and tailored interventions for stress reduction for those requiring further support should be investigated. Additionally, an improved understanding of the cortical and subcortical pathways driving the cardiac response under mental stress should be further investigated with the help of sophisticated neuroimaging methods. The latter may lead to the implementation and standardisation of mental stress tests in clinical practice as a tool to evaluate individual risk of developing AF.

## Conclusion

AF is the most common form of cardiac arrhythmia and its increasing incidence is a worldwide burden. Mental stress has been shown to impact the cardiovascular system and to induce changes that can lead to the onset of AF. Negative emotions such as anger and anxiety and depressive symptoms have also been linked to an increased risk of developing AF in several observational studies, with their role as potential trigger of AF events suggested by laboratory-induced stress test and few observational studies. Psychological stress and emotions affect blood flow (increased heart rate and hypertension) and directly affect the heart by inducing alterations in cardiac electrical activity, which may lead to cardiac arrhythmias. Unfortunately, screening for mental health and psychological stress are not currently embedded into clinical pathways for the management of AF. However, the role that psychological stress and emotions on the onset of AF and on its recurrence need to be taken into consideration, as they can affect the management of these patients. AF has shown to be a preventable disease and helping in mange the emotional response and reducing mental stress may be effective in reducing the incidence of the disease.
